# The Quality of Life in Patients With Valve Prosthesis After Undergoing Surgery for Valvular Heart Diseases

**DOI:** 10.7759/cureus.43030

**Published:** 2023-08-06

**Authors:** Khalid E Al-Ebrahim, Shomokh A Albishri, Sarah W Alotaibi, Lama A Alsayegh, Ebtesam M Almufarriji, Raghad B Babader, Shahad A Abdulgader, Alaa A Alsaegh, Rami S Alghamdi, Ahmed A Elassal

**Affiliations:** 1 Cardiac Surgery Unit, Department of Surgery, King Abdulaziz University Hospital, King Abdulaziz University, Jeddah, SAU; 2 Department of Cardiac Surgery, King Abdullah Medical City, Makkah, SAU; 3 Department of Cardiothoracic Surgery, Zagazig University, Zagazig, EGY

**Keywords:** king abdulaziz university hospital (kauh), jeddah saudi arbia, cross sectional studies, surgical replacement of valve, valve prosthesis, valvular heart diseases, quality of life

## Abstract

Background and objective

Surgery for valvular heart disease by valve replacement procedures has become one of the most frequently performed cardiac operations to improve the quality of life (QoL). Its long-term outcomes are assessed using the quality-of-life index (QLI). This study aimed to evaluate the QoL in patients who received valve prostheses after surgery for valvular heart diseases at King Abdulaziz University in Jeddah from 2010 to 2023.

Methods

This was a descriptive cross-sectional study of 59 patients aged 18 years or older who underwent surgical mitral and aortic valve replacement, involving either mechanical or tissue valves, from January 2010 to May 2023 They were selected using a non-probability convenient sampling technique. Their medical records were reviewed and the participants were interviewed via phone using the World Health Organization Quality of Life-BREF (WHOQOL-BREF) questionnaire, which was used to measure the QoL of patients (https://neurotoolkit.com/whoqol-bref/).

Results

The study found that the QoL of the participants varied across different domains. The psychological domain had the highest mean score of 79.76, while the physical domain had the lowest mean score of 61.5. The other domains, - social, environmental, and spiritual - had mean scores of 68.05, 69.9, and 73.25, respectively. There was a statistically significant association between the QoL and nationality and chronic diseases. However, the duration after surgery and the type of valve did not significantly correlate with the QoL in the different domains.

Conclusion

Based on our findings, heart valve replacement improves the QoL of patients. Healthcare organizations and providers should aim to improve the management of chronic diseases to optimize outcomes.

## Introduction

According to the World Health Organization (WHO), health refers to the absence of disease or infirmity, as well as a state of complete physical, mental, and social well-being. Moreover, health-related quality of life (HRQol) comprises various aspects of physical, mental, emotional, and social functioning [1]. Cardiovascular disease (CVD) is the most common cause of death worldwide and encompasses any condition that affects the heart muscle, valves, rhythm, or blood vessels. Furthermore, valvular heart disease is expected to remain a leading contributor to cardiovascular morbidity and mortality in the coming decades. Among all deaths resulting from valvular heart disease, aortic valve diseases account for 61% of cases, while mitral valve diseases account for 15%.

The field of heart valve replacement surgery has witnessed significant advancements over the past three decades, becoming one of the routine cardiac treatments to improve QoL [2]. The replacement of the affected valve can be performed using mechanical and biological valves. The long-term effects of valve replacement surgery on the quality-of-life index (QLI)-determining factors include returning to regular daily activities, maintaining self-esteem, and frequent interactions at work, in the community, and at home [3]. Several studies have shown that patients who survive the surgery and postoperative period have a similar QoL, function, and survival as their age-matched peers in the general population, and valve-related complications occur at an acceptable rate after cardiac valve replacement [4]. The primary objective of this study is to determine if undergoing valve replacement surgery enhances a patient’s QoL and identify the factors that can accurately predict this outcome. The functional status and changes in the QoL of patients who have undergone valvular replacement surgeries have not yet been adequately assessed among the Saudi population. Most cardiac-related research focuses on studying physical operative results, surgical complications, and prophylactic and management protocols while QoL has not received similar attention [5-8]. In light of this, our study aims to assess the QoL of patients at King Abdulaziz University, who have received prosthetic heart valve replacements.

## Materials and methods

This was a descriptive cross-sectional study involving 59 individuals who underwent valve replacement surgery. This research was authorized by the Institutional Review Board of King Abdulaziz University Hospital (Ref: 283-23). Data collection commenced in May 2023 and continued for one month, utilizing a non-probability convenient sampling technique. The patients were selected from the cardiac surgery list of individuals who underwent valve replacement surgery at King Abdulaziz University Hospital between January 2010 and May 2023. The medical records of the selected participants were reviewed, and they were interviewed via telephone. We explained the study's purpose, obtained their verbal consent, and informed them that their responses would remain confidential. We included all patients aged 18 years or older who underwent surgical mitral and aortic valve replacement, involving either mechanical or tissue valves. A total of 158 patients underwent mitral or aortic valve replacement surgery; however, we excluded 99 patients, including those who had died (n = 14), changed phone numbers (n = 21), did not reply to phone calls (n = 45), refused to participate in the study (n = 12), or did not have their phone numbers documented in their files (n = 7). Additionally, patients who had undergone valve repair surgery, coronary artery bypass surgery, or repair of a congenital heart defect were excluded.

The data collected through the survey involved two sections. The first section consisted of demographic data, chronic diseases including hypertension, diabetes mellitus, chronic kidney disease, liver disease, osteoporosis, and others, and any complications that occurred after the valve replacement surgery, including heart failure, pleural effusion, coagulopathy, valve thrombosis, stroke, infective endocarditis, renal failure, or valve dysfunction. In the second section, the questionnaire was used to measure the QoL of patients who underwent valve replacement surgery. In our study, we applied the World Health Organization QOL-BREF (WHOQOL-BREF; https://neurotoolkit.com/whoqol-bref/) [9]. Each question in the questionnaire was scored from 1 to 5 on the response scale; the scores were then extrapolated to a 0-100 scale for each domain based on each participant’s response.

The data were entered into a Microsoft Excel sheet, and statistical analysis was conducted using IBM SPSS Statistics version 26 (IBM Corp., Armonk, NY). We calculated the mean and standard deviation (SD) for some of the variables, while frequencies and percentages were used for other variables. We employed the independent t-test to compare two groups and one-way ANOVA to compare three groups or more. Statistical significance was set at p<0.05.

## Results

The study population consisted of 59 participants, all aged 18 years or older, with a mean age of 47.64 (SD = 16.57) years. Of them, 34 (57.6%) were male and 25 (42.4%) were female. Most participants were non-Saudi (n = 44, 74.6%) and had a normal BMI of 22 kg/m^2^ (37.3%). Almost half of the patients had undergone aortic valve replacement (n = 29, 49.2%). A majority of the patients were non-smokers (n = 51, 86.4%), had more than one year of duration after surgery (n = 58, 98.3%), and had a mechanical type of valve (n = 41, 69.5%). Table 1 shows the demographic characteristics of the patients.

**Table 1:** Demographics and patient characteristics BMI: body mass index

Variable		N (%)
Gender	Male	34 (57.6%)
	Female	25 (42.4%)
Nationality	Non-Saudi	44 (74.6%)
	Saudi	15 (25.4%)
BMI	Normal	22 (37.3%)
	Overweight	19 (32.2%)
	Underweight	6 (10.2%)
	Extremely obese	12 (20.3%)
Smoking status	No	51 (86.4%)
	Yes	8 (13.6%)
Duration of time after surgery	Less than 1 year	1 (1.7%)
	More than 1 year	58 (98.3%)
Valve replaced	Mitral	26 (44.1%)
	Aortic	29 (49.2%)
	Mitral and aortic	4 (6.8%)
Type of valve	Tissue	14 (23.7%)
	Mechanical	41 (69.5%)
	Tissue and mechanical	1 (1.7%)
	N/A	3 (5.1%)

Table 2 shows the association of all variables with the overall QoL; nationality displayed a significant association (p<0.05), where the Pearson correlation coefficient (r = 0.35) had a weak positive correlation. There was also a statistically significant difference in terms of chronic diseases, as the Pearson correlation coefficient (r = -0.5) moderated the negative correlation.

**Table 2:** Association between variables and overall quality of life *Statistically significant BMI: body mass index; SD: standard deviation

Variable		Quality of life score, Mean ±SD	P-value
Gender	Male	71.83 ±16.85	0.829
	Female	72.3 ±12.45	
Nationality	Non-Saudi	69.41 ±15.35	0.019*
	Saudi	79.84 ±11.15	
BMI	Normal	75.06 ±13.05	0.615
	Overweight	70.06 ±14.45	
	Underweight	73.83 ±12.65	
	Extremely obese	68.84 ±20.31	
Surgical complication	No	72.50 ±13.86	0.700
	Yes	70.75 ±18.50	
Chronic disease	No	78.34 ±12.04	0.00*
	Yes	63.52 ±14.64	
Smoking status	No	72.04 ±14.51	0.879
	Yes	72.20 ±19.16	
Duration of time after surgery	Less than 1 year	75.00 ±0.00	0.846
	More than 1 year	72.01 ±15.15	
Valve replaced	Mitral	72.11 ±16.18	0.88
	Aortic	71.54 ±14.14	
	Mitral and aortic	75.51 ±17.26	
Type of valve	Tissue	78.06 ±9.61	0.366
	Mechanical	70.52 ±14.91	
	Tissue and mechanical	70.25 ±0.00	
	N/A	65.66 ±33.73	

**Figure 1:**
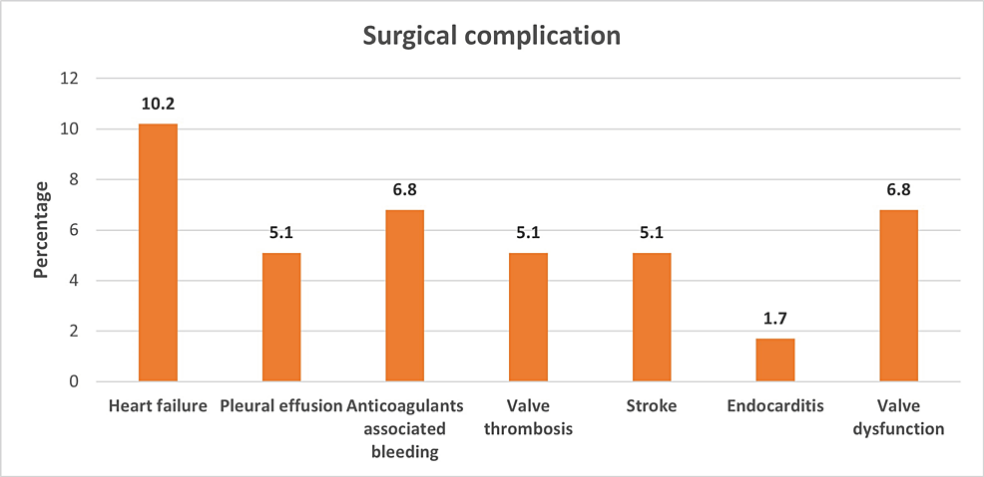
Proportion of surgical complications among the patients

**Figure 2:**
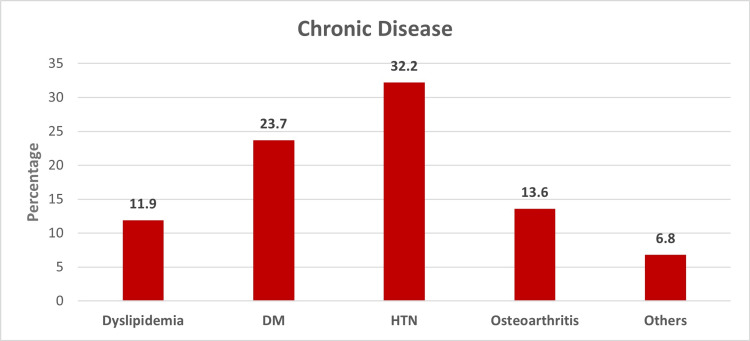
Proportion of chronic diseases among the patients DM: diabetes mellitus; HTN: hypertension

**Figure 3:**
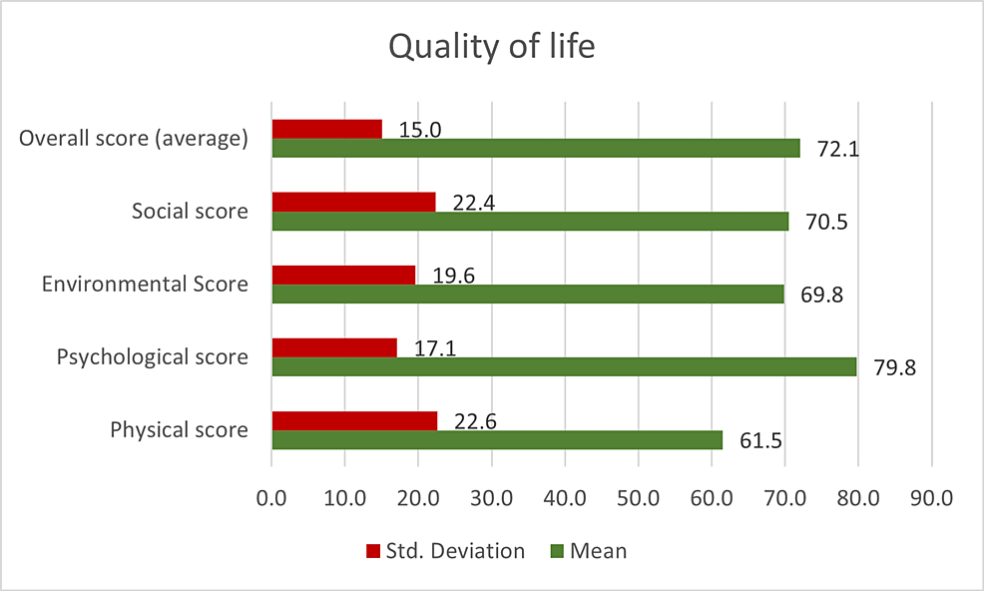
Mean scores and standard deviations for each domain of quality of life and the overall average

Figure 1 displays the proportion of various surgical complications (25.4% overall) among the patients. Heart failure and anticoagulant-associated bleeding were the most common complications, occurring in 10.2% and 6.8% of patients, respectively. Figure 2 shows the proportion of various chronic diseases in the cohort (42.4% overall). Hypertension was the most common chronic disease, occurring in 32% of patients. Figure 3 displays the mean score for each domain of quality of life and the overall average. The psychological domain had the highest mean score (79.76), while the physical domain had the lowest (61.5).

## Discussion

Several studies have compared biological and mechanical prosthetic valves in terms of mortality, morbidity, frequency of reoperations, and bleeding issues; however, patient QoL has never been the primary focus of these studies [5-8,10]. Patients may experience physical, social, emotional, or psychological side effects from valve replacement surgery even in the absence of surgical complications. Staying in the hospital might affect both personal and professional lives. Moreover, administrative and financial burdens might persist even beyond the hospital stay. Patients’ perceptions of their QoL following valve replacement surgery may be influenced by physical restrictions, discomfort, and the presence of scars [11]. An increasing number of older patients are undergoing open heart surgery. Heart surgery has less of an impact on future morbidity or life expectancy in older patients, but symptom alleviation and better overall functional ability may be more significant to such patients [10]. A previous study was conducted in the western region of Saudi Arabia at the King Faisal Cardiac Center at King Abdul-Aziz Medical City. The mean age of the participants was 60 years. More than two-thirds of participants had a history of ischemic heart disease (IHD). Coronary artery bypass graft (CABG) was the most common cardiac surgery performed (80.9%). According to the findings, patients' QoL was significantly impacted after cardiac surgery. Based on the 36-Item Short Form Health Survey (SF-36) questionnaire, out of the eight QOL domains, the physical functioning subscale, role limits caused by physical problems, and role limitations resulting from emotional problems had the lowest means. The subscales measuring pain, mental health, and emotional well-being had the highest scores overall [2].

In 2021, the Nepal Institute of Development Studies carried out a cross-sectional descriptive study involving 131 rheumatic heart disease patients who had undergone heart valve replacement surgery. Prior to and following surgery, the QoL was evaluated using the Ferrans and Powers QLI (cardiac version IV) via a questionnaire. The majority of the patients underwent mitral valve replacement surgery. The total improvement observed in percentages was as follows: 28.62% in overall QLI, 47.15% in the health and functional domain, 16.68% in the psychological and spiritual domain, 26.24% in the social and economic domain, and 11.49% in the family domain, which represents the most significant improvement across all other categories. The findings also imply that family support appears to be the population's greatest strength [6]. The demographic characteristics of the participants in our study showed no significant correlation with the total QoL score, except for nationality (p = 0.019), which showed a weak positive correlation, and chronic diseases (p = 0.0001), which showed a negative correlation. Saudi patients have better postoperative QoL mainly because of better family, socioeconomic, and financial support.

The negative correlation in terms of chronic diseases is the burden of multiple follow-ups, investigations, and admissions. In this study, there were no significant differences with regard to gender. This finding is in line with that of Aljafari et al. [2]. On the other hand, some other studies [12-13] have reported better QoL among males compared to females. There are several factors that may contribute to the poorer QoL that some women experience after cardiac surgery compared to men. These factors include age: women tend to have cardiac surgery at an older age than men, which may increase the risk of complications and reduce the overall QoL after surgery. Also, women are more likely to present with comorbidities before surgery, such as diabetes, hypertension, and obesity, which can result in lower QOL scores at baseline and increase the risk of surgical complications. Hormonal differences between men and women may also play a role. For example, estrogen has been shown to have cardioprotective effects, and women may experience a decline in estrogen levels after menopause, which could contribute to poorer outcomes after cardiac surgery. Women may also face additional social and cultural barriers to recovery after cardiac surgery, such as a lack of support or access to resources. Moreover, women may experience more intense fear pre and postoperatively than men, and they may be less knowledgeable about how cardiac disease develops. Fears relating to anesthesia, pain, and anxiety about postoperative recovery can also affect the QOL score. Finally, it has been found that women experienced depressive symptoms before hospital discharge and throughout follow-up [13]. We found no significant correlation between postoperative complications and QoL, which can be explained by the small sample size of our study. Therefore, a significant correlation between complications and QoL could not be detected. The severity of postoperative complications may also influence the relationship between complications and QoL. If the complications are relatively mild and easily managed, they may have less of an impact on QoL than more severe complications. This finding is consistent with another study [2]. Also, Kurfirst et al. found that there was no significant correlation between postoperative complications and QOL, except for body pain, among patients of advanced age (>70 years) [14].

Our findings showed that chronic diseases have a significant impact on QoL. Diabetes and hypertension may be associated with a higher risk of developing complications after valve replacement surgery. These complications can include infections, bleeding, or problems with the heart or other organs, which can impact QoL. Moreover, they can also lead to emotional distress such as anxiety or depression, which can impact QoL as well. Patients may worry about their health, their ability to manage their disease, or their future prospects, which can impact their overall well-being. Similarly, chronic diseases were the leading cause of death in Iran in 2012. Also, cardiovascular disorders were the most prevalent type of disease. The patients had very high rates of diabetes, hypertension, and IHD. Globally, these three conditions have been regarded as the biggest risk factors for CVD [15-17]. Unfortunately, these variables have become more common in the Saudi community because of the widespread adoption of a Westernized lifestyle and food habits, necessitating prophylactic measures in primary healthcare and educational settings [18-21].

Most chronic diseases can affect patients' general health, limit their performance, diminish their HRQoL, and increase their healthcare costs since they limit their ability to perform activities of daily life [22-23]. According to WHO data, the rate of increase in chronic diseases reached a very high level in 2002-2003, and it is predicted that by 2020, issues caused by chronic diseases will account for approximately 80% of the global burden of disease in developing countries [22]. Many studies have found that diabetes mellitus has a negative effect on QoL [19-21]. Significant correlations between QoL dimensions and conditions like diabetes and hypertension have been found. Since these conditions have become common in the Saudi Community, changing one's lifestyle before and after cardiac surgery will enhance the QoL [18]. According to a study released after 2000, the prevalence of problems connected to valves ranges from 0.7% to 3.5% per patient year [24-26]. In our study, surgical complications did not affect QoL while it was an important factor in other studies; this difference could be attributed to differences in the study population in terms of age, comorbidities, and severity of illness. Also, the implementation of prophylactic and management protocols and road maps can help minimize and properly address these complications [24-29]. Family support seems to be an important factor with regard to patient health in Saudi patients when compared to their non-Saudi counterparts [30].

Preoperative family support is crucial to avoid psychological and physical cardiac surgery-related complications like wound infection, cardiovascular events, readmissions, and mortality [31-32]. The Saudi government sponsors a wide range of social services programs aimed at ensuring that every citizen has a decent standard of living and improving the quality of medical care and services. Cardiac surgery outcomes, including those related to valve replacement surgery, have been shown to be generally favorable in Saudi Arabia. The availability of specialized cardiac centers and experienced surgeons, who are well-trained either locally or internationally, contributed to these outcomes [33]. In terms of lifestyle factors, "Saudi Arabia Initiatives to Promote a Healthy Lifestyles" provides an overview of the various initiatives and programs that have been implemented in Saudi Arabia to promote healthy lifestyles, including physical activity and healthy eating [34]. The field of cardiac valve surgery is constantly evolving, and there are several ongoing research efforts aimed at improving outcomes and QoL for patients, including using minimally invasive and transcatheter interventions [35].

Limitations of the study

This study has a few limitations, primarily its small sample size and the fact that this was a single-center study. Poor patient response to the questionnaire is another major limitation.

## Conclusions

Based on our findings, the QoL of patients who undergo surgical valve replacement procedures has improved overall. Worldwide measures are needed to control and manage major risk factors that cause chronic diseases. There was no significant association between QoL and surgical complications. The patient’s age, gender, type of valve, and surgical complications did not affect QoL in patients who underwent valve replacement. Further studies with a more representative sample should be conducted to validate these findings.
